# Fetal Neuroimaging in Aicardi Syndrome: A Case Report and Literature Review

**DOI:** 10.7759/cureus.101877

**Published:** 2026-01-19

**Authors:** Imen Bannour, Ekram Guerbej, Hafedh Abbassi, Sassi Boughizane

**Affiliations:** 1 Department of Obstetrics and Gynecology, Faculty of Medicine, Ibn El Jazzar of Sousse/Farhat Hached University Hospital, Sousse, TUN

**Keywords:** aicardi syndrome, corpus callosum agenesis, fetal mri, fetal ultrasound, prenatal diagnosis

## Abstract

Aicardi syndrome (AIC) is a congenital condition involving characteristic neurological and ocular abnormalities. We describe a 30-year-old pregnant woman referred at 32 weeks of gestation after an ultrasound at 29 weeks showed corpus callosum agenesis, an interhemispheric arachnoid cyst, and colpocephaly. These findings raised prenatal suspicion of AIC. Postnatal evaluation confirmed the diagnosis with the identification of chorioretinal lacunae on fundoscopic examination. In this case, the prenatal imaging findings were key to recognizing a pattern suggestive of AIC, while postnatal assessment provided the definitive confirmation. This report highlights the role of detailed fetal neuroimaging in raising suspicion of AIC during pregnancy and underscores the importance of coordinated postnatal evaluation and multidisciplinary counseling.

## Introduction

Aicardi syndrome (OMIM: 304050; AIC) is a rare and severe neurodevelopmental disorder first described in 1965 by French neurologist Dr. Jean Aicardi [1]. It is a congenital syndrome defined by the classical triad of total or partial corpus callosum (CC) agenesis, chorioretinal lacunae (CRL), and epileptic infantile spasms [2]. Since 2005, diagnosis criteria have been revised to encompass a broader range of features, owing to advances in modern imaging [3]. Although the diagnosis of AIC is based solely on clinical features, it is now seen as a heterogeneous disorder involving neurological and somatic manifestations beyond the classical triad. Consequently, additional supportive features have been incorporated into the diagnostic criteria [3,4]. AIC is a rare disease with an estimated incidence ranging from one per 93,000 to 167,000 live births [5]. The disorder is reported to affect only females or males with a 47,XXY karyotype [6]. This observation suggests that AIC was an X-linked, male-lethal disorder [3,6]. Because diagnostic criteria cannot be fulfilled prenatally, since seizures and CRL can only be observed postnatally, only a few cases of prenatal diagnosis have been reported worldwide [7,8].

Herein, we report a case of AIC suspected in a female fetus at 29 weeks of gestation, which was confirmed postnatally.

## Case presentation

**Figure 1:**
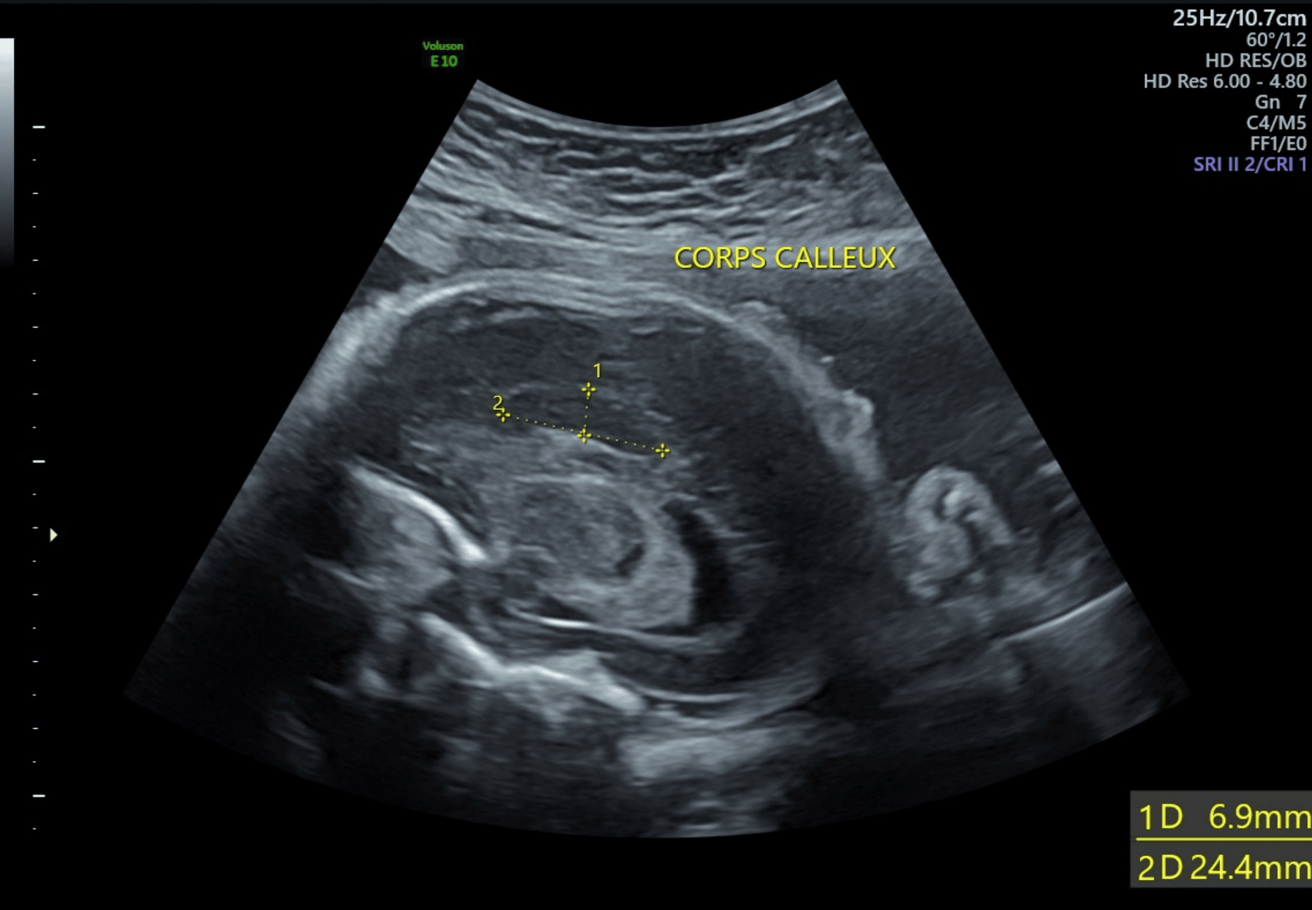
Sagittal section of the fetal brain showing the partial agenesis of the corpus callosum

**Figure 2:**
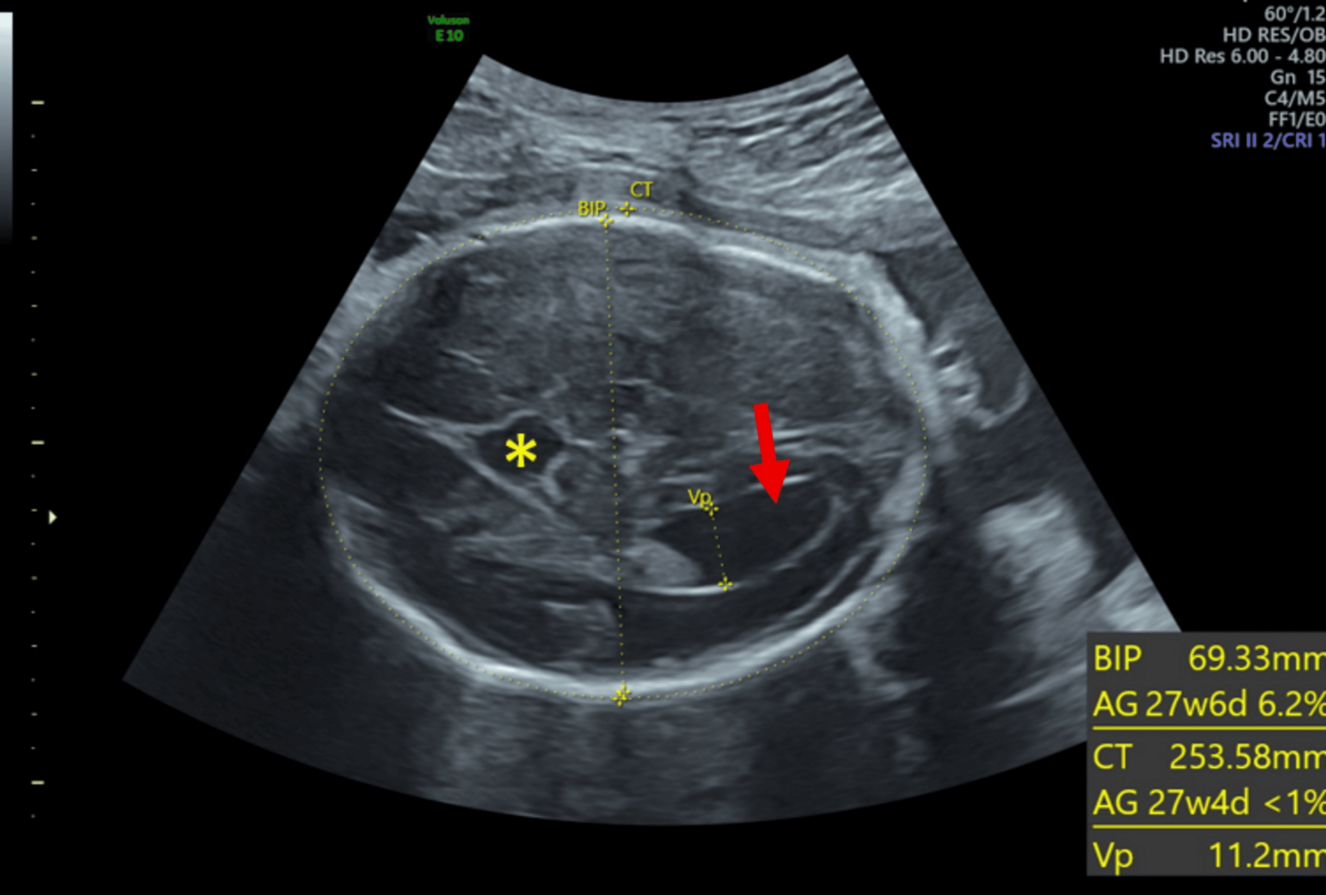
Axial section of the fetal brain showing a colpocephaly (arrow) and a widening of the interhemispheric fissure and arachnoid cyst (asterisk)

A 30-year-old pregnant woman was referred to our tertiary center at 32 weeks of gestation following the detection of multiple cerebral abnormalities on a fetal sonogram performed at 29 weeks. She was a gravida 2, para 1. Her first pregnancy was uneventful, resulting in the delivery of a healthy term infant two years prior. The patient had no relevant family history, and the marriage was non-consanguineous. Due to socioeconomic constraints, the patient did not undergo the recommended first and second trimester ultrasound (US) examinations, which explains the relatively late discovery of fetal malformations. Ultrasound revealed a female fetus with a 20 mm interhemispheric arachnoid cyst, a short and thick dysgenetic corpus callosum (Figure 1), bilateral colpocephaly measuring 12 mm, and widening of the interhemispheric fissure (Figure 2).

**Figure 3:**
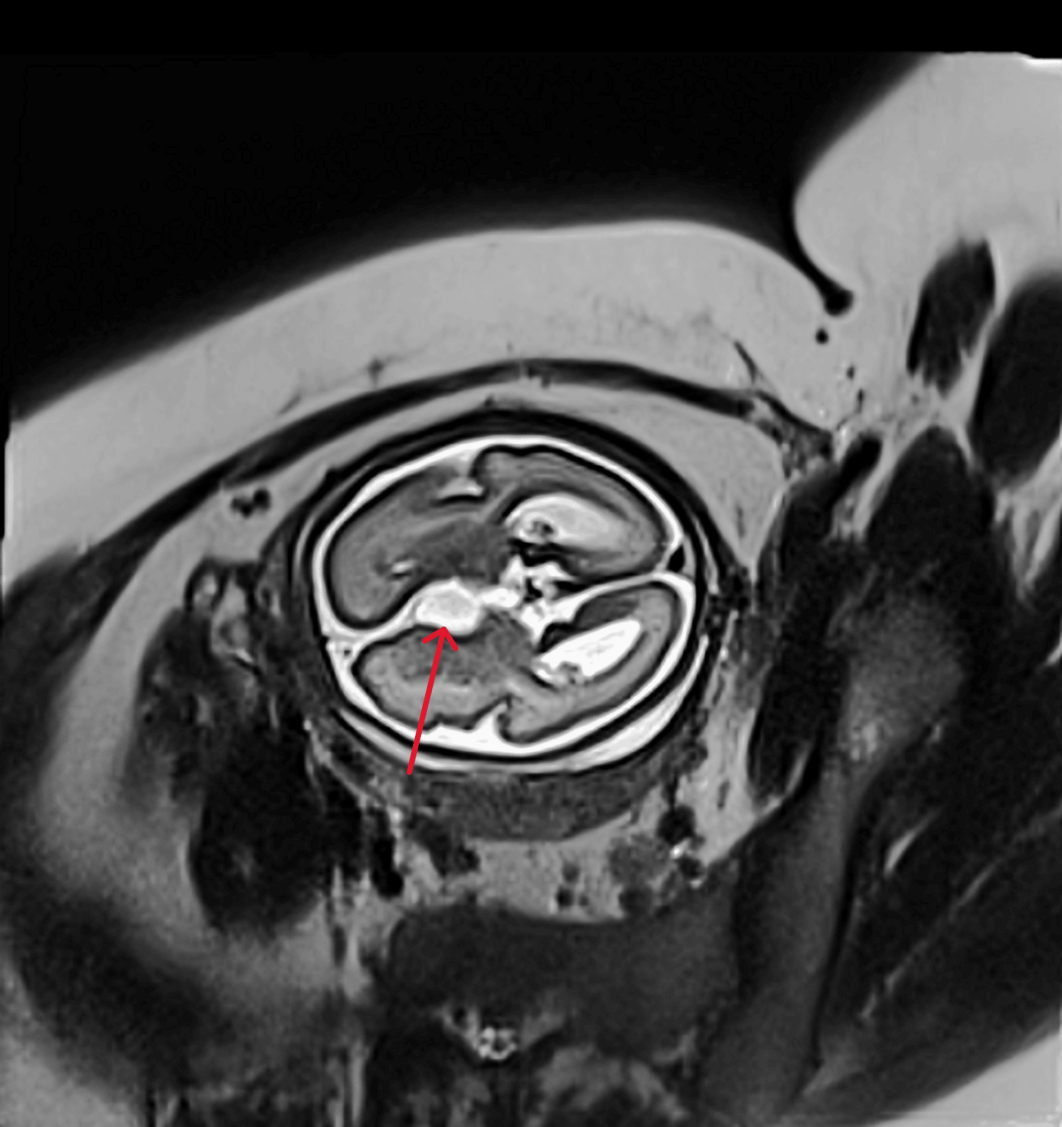
Fetal MRI: T2 sequence showing an interhemispheric arachnoid cyst (arrow)

The remainder of the US examination was unremarkable, with no skeletal or vertebral anomalies and normal growth parameters. At this stage, AIC was suspected. To further assess the malformations, fetal magnetic resonance imaging (MRI) was performed. MRI findings confirmed our initial suspicion, revealing widening of the interhemispheric fissure with asymmetry of its borders in the frontal region. An interhemispheric cyst measuring 18 mm in its longest axis was identified (Figure 3), showing a T2 hyperintensity and T1 hypointensity with well-defined margins.

**Figure 4:**
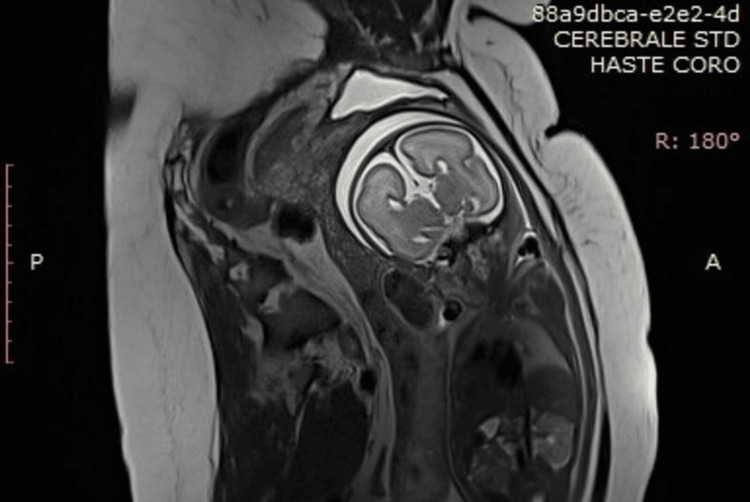
Fetal MRI in coronal T2 sequence showing the widening of the frontal lobes exhibiting a “bull’s horn” configuration

**Figure 5:**
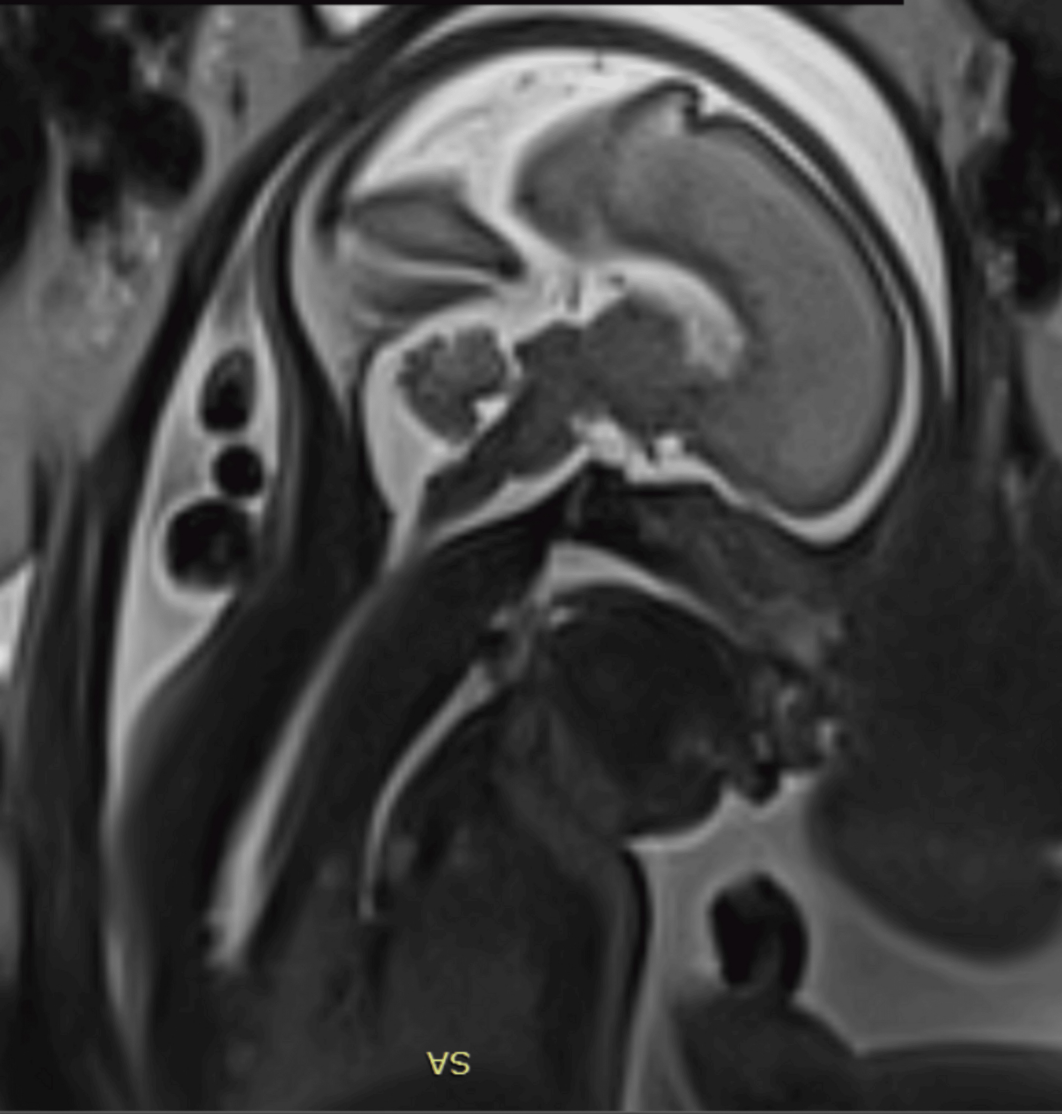
Fetal MRI: T2 sequence showing the absence of the corpus callosum

Complete agenesis of the CC was noted, with widening of the frontal horns exhibiting a “bull’s horn” configuration, associated with bilateral colpocephaly measuring 10 mm at the atria of the lateral ventricles (Figures 4, 5). Hypoplasia of the Sylvian fissure was also observed. Termination of pregnancy was not considered due to the advanced gestational age and Tunisian regulations. Labor was induced at 39 weeks of gestation, and the patient delivered a female infant, weighing 3200 g, with Apgar scores of 8, 9, and 10 at one, five, and 10 minutes, respectively. The newborn was immediately admitted to the neonatal intensive care unit (NICU), and early follow-up was uneventful. An ophthalmic examination was scheduled on the fifth day of life. Funduscopic examination revealed chorioretinal lacunae, confirming the diagnosis of AIC.

## Discussion

AIC is a very rare congenital disorder. Based on an international survey of AIC cases, the prevalence has been estimated at over 4,000 cases worldwide [3]. A Norwegian epidemiological study reported an age-adjusted prevalence of 0.63 per 100,000 females [5]. The rarity of this syndrome results in scarce literature and makes diagnosis challenging for clinicians. Furthermore, with no known etiology, the diagnosis of AIC is based solely on clinical features [3]. Initially, AIC was proposed to be an X-linked genetic disorder, presenting almost exclusively in female patients. Only a few cases of males with a 47,XXY karyotype have been reported [4]. The prevailing hypothesis is that AIC is caused by a de novo mutation in one of the X chromosomes and inherited in a dominant manner with hemizygous lethality in males [5]. Indeed, when the mutation occurs in a male, it affects the single available X chromosome, resulting in lethality during early development in male conceptuses [1,9]. In our case, the fetus was female on US examination, which is consistent with the diagnosis of AIC.

In 1965, AIC was defined by the classical triad of CC agenesis, CRL, and infantile spasms. In 2005, Sutton et al. proposed that the presence of all three classical features is diagnostic for AIC and that the presence of two classical features in addition to at least two major (cortical malformations, periventricular and subcortical heterotopia, intracranial cysts around the ventricle and/or choroid plexus, optic disc/nerve coloboma or hypoplasia) or supporting features (“split-brain” EEG, gross cerebral hemispheric asymmetry, microphthalmia, vertebral and costal abnormalities, prominent premaxilla, vascular malformations, or vascular malignancy) is strongly suggestive of an AIC diagnosis [4]. This updated approach highlights the pleiotropic nature of AIC. In our case, the suspicion was based on CC agenesis and intracranial cysts, with the diagnosis confirmed postnatally by the presence of CRL on funduscopic examination. CRL remains the most consistently observed ocular feature in affected patients. However, other ocular abnormalities, such as coloboma and microphthalmia, can also fulfill the diagnostic criteria when CRLs are absent, as long as typical seizure types and characteristic malformation patterns are present [6].

Since key diagnostic features such as CRL and spasms cannot be observed in the prenatal phase, AIC is infrequently diagnosed before birth, which may explain the scarcity of prenatally diagnosed cases reported in the literature [7]. With the improvement of imaging techniques, AIC diagnosis is now based on the classical triad and major radiological features observed on US and MRI. Table 1 summarizes selected reports of prenatal and postnatal imaging features in AIC.

**Table 1:** Literature review table of selected reports describing imaging features of Aicardi syndrome US: ultrasound; MRI: magnetic resonance imaging; CCA: corpus callosum agenesis

Study (Year)	Type of study	Population number	Sex	Imaging modality	Prenatal/Postnatal	Imaging findings
Curatolo et al. (1980) [10]	Case report	1	Male	Tomography	Postnatal	CCA
Glasmacher/Sutton et al. (2007) [11]	Survey	69	68 females, 1 male (47, XXY)	US and MRI	Postnatal	CCA, intracranial cysts, cortical malformations (periventricular heterotopia, polymicrogyria)
Masnada et al. (2020) [2]	Prenatal MRI case series	9	Predominantly females	Prenatal and postnatal MRI ± US	Prenatal	Interhemispheric fissure distortion, extra-axial cysts, Partial/Complete CCA
Pomar et al. (2022) [7]	Retrospective series	20	Females	US and MRI	US and MRI (Prenatal) with postnatal confirmation	Interhemispheric fissure distortion, extra-axial cysts, Partial/Complete CCA; Frontal asymmetry frequent
Bromley et al. (2000) [12]	Prenatal case reports	2	Females	US	Prenatal	Ventriculomegaly, CCA, interhemispheric cyst
Cabrera et al. (2011) [13]	MRI cohort	26	Females	MRI	Postnatal	Cerebral lesion laterality (right-sided predominance)

In an international survey conducted between 2000 and 2020, Pomar et al. analyzed 20 cases of prenatally suspected and postnatally confirmed AIC and concluded that most malformations identified on postnatal radiological examination can be detected prenatally [7]. In their study, the authors observed that arachnoid cysts associated with distortion of the interhemispheric fissure were constantly encountered. This finding was also reported by Silvia et al. in their review, where cerebral cysts were identified as the main feature associated with CC dysgenesis [2]. In our case, both fetal US and MRI concluded to CC agenesis resulting in the “bull’s horns” image, associated with an arachnoid cyst in the interhemispheric fissure. These findings alone justified our suspicion of AIC. Neuronal migration anomalies are also reported in AIC [3]. Hypoplasia of the Sylvian fissure is one such cortical malformation that we observed in our fetus. This was consistent with Pomar et al., who found these anomalies in 88% of their cases [7]. In our case, bilateral colpocephaly was observed, corresponding to selective enlargement of the posterior lateral ventricles. Ventriculomegaly, frequently reported in AIC, usually reflects the same posterior horn dilation associated with CC agenesis [2,7]. In some reported cases of AIC, vertebral and skeletal malformations can be observed, including hemivertebrae, butterfly vertebrae, or rib fusion [3,9]. In our case, both fetal US and MRI examinations showed no additional abnormalities, highlighting the heterogeneity of AIC.

## Conclusions

AIC is a rare neurodevelopmental disorder, often associated with a poor prognosis. In the absence of a specific etiology, it poses significant diagnostic challenges, particularly in the prenatal period, when clinical features are lacking, and diagnosis relies solely on radiological findings. Our case highlights the crucial role of detailed fetal neuroimaging in improving early recognition and guiding parental counseling.
